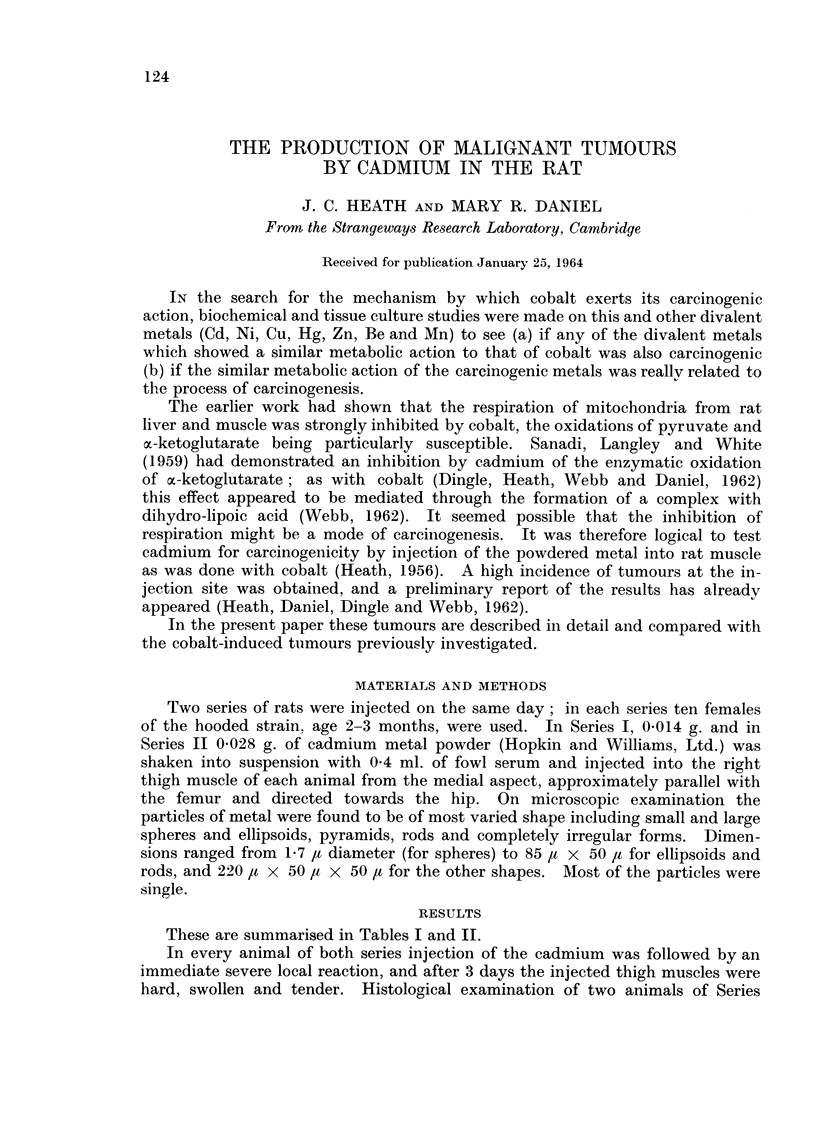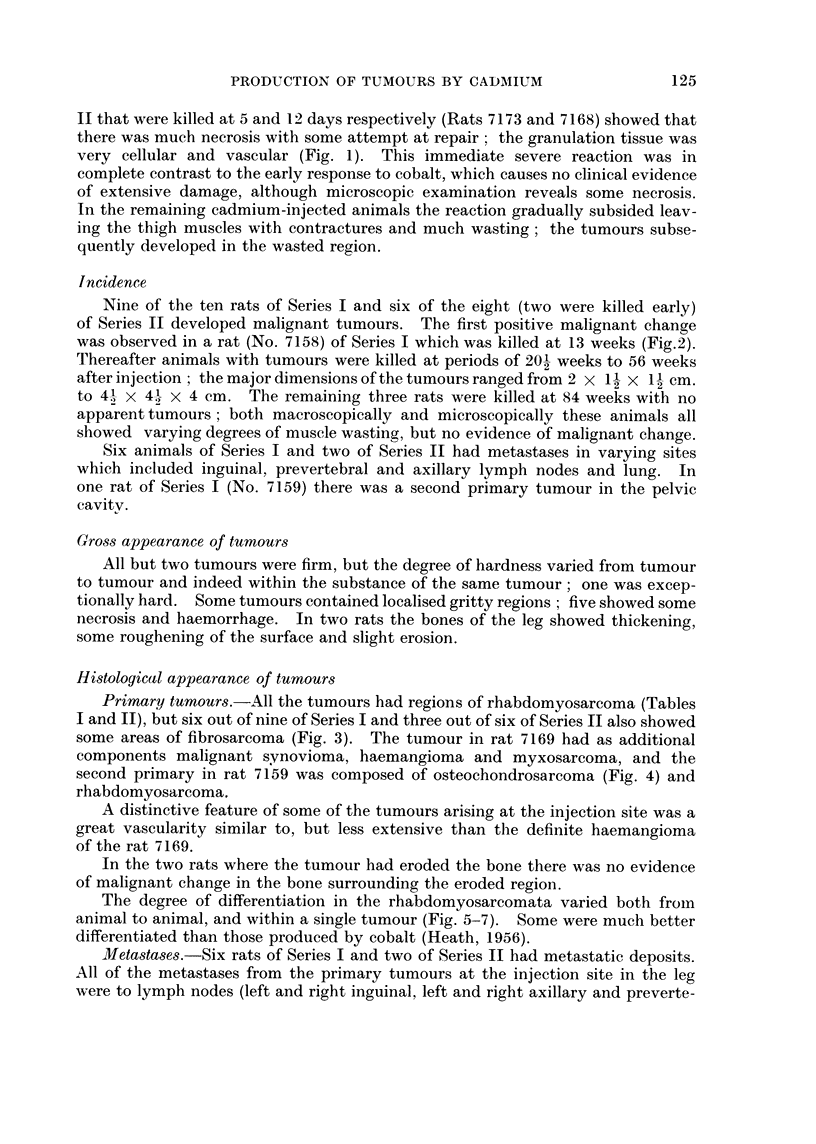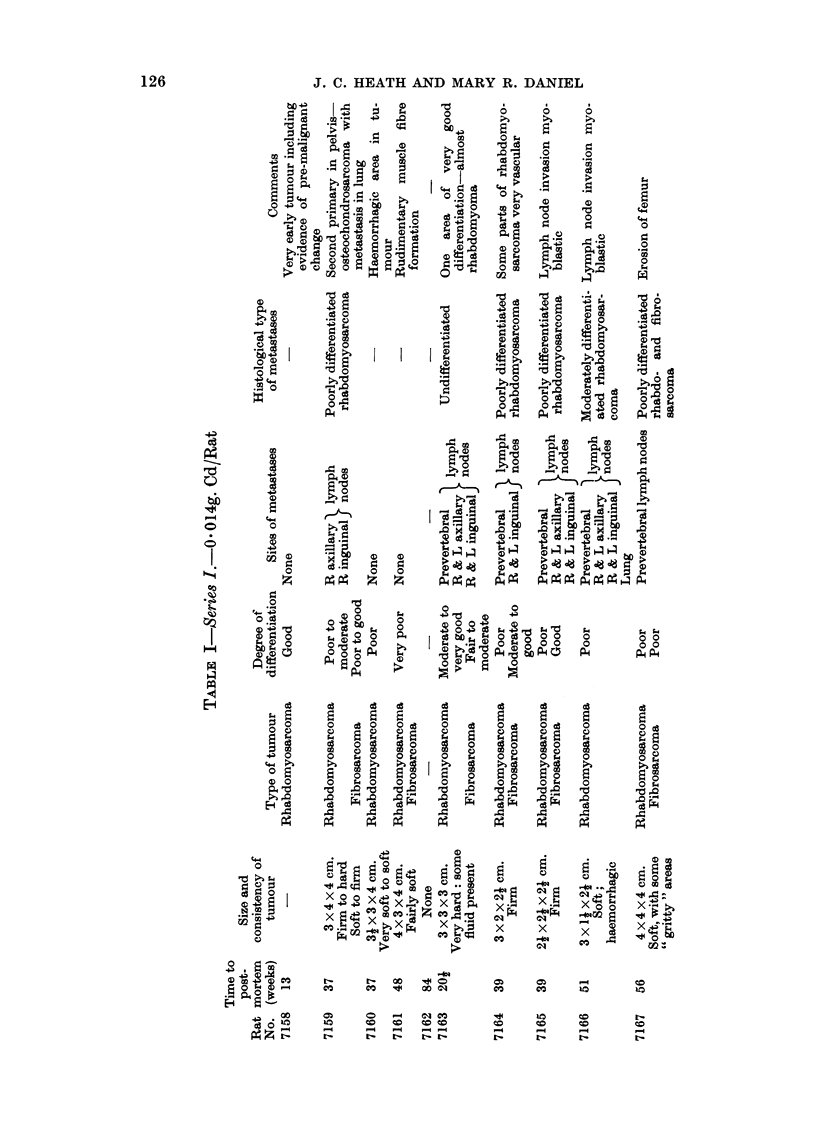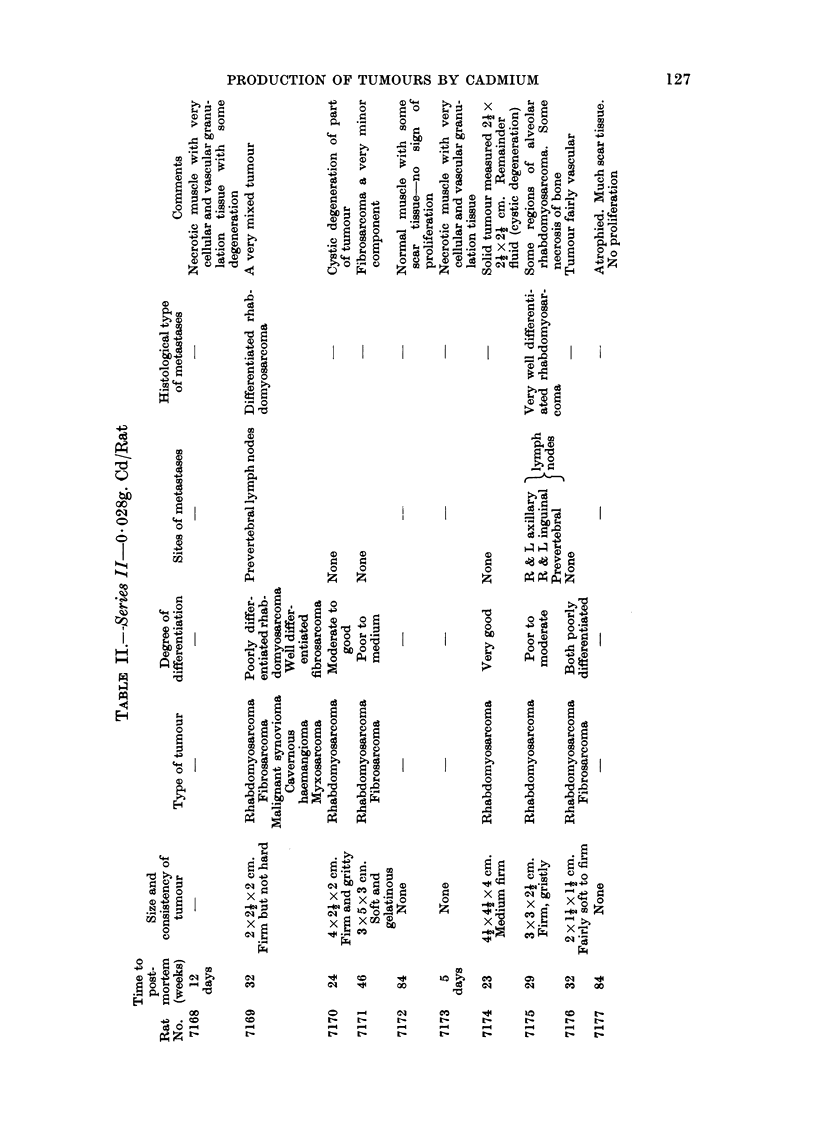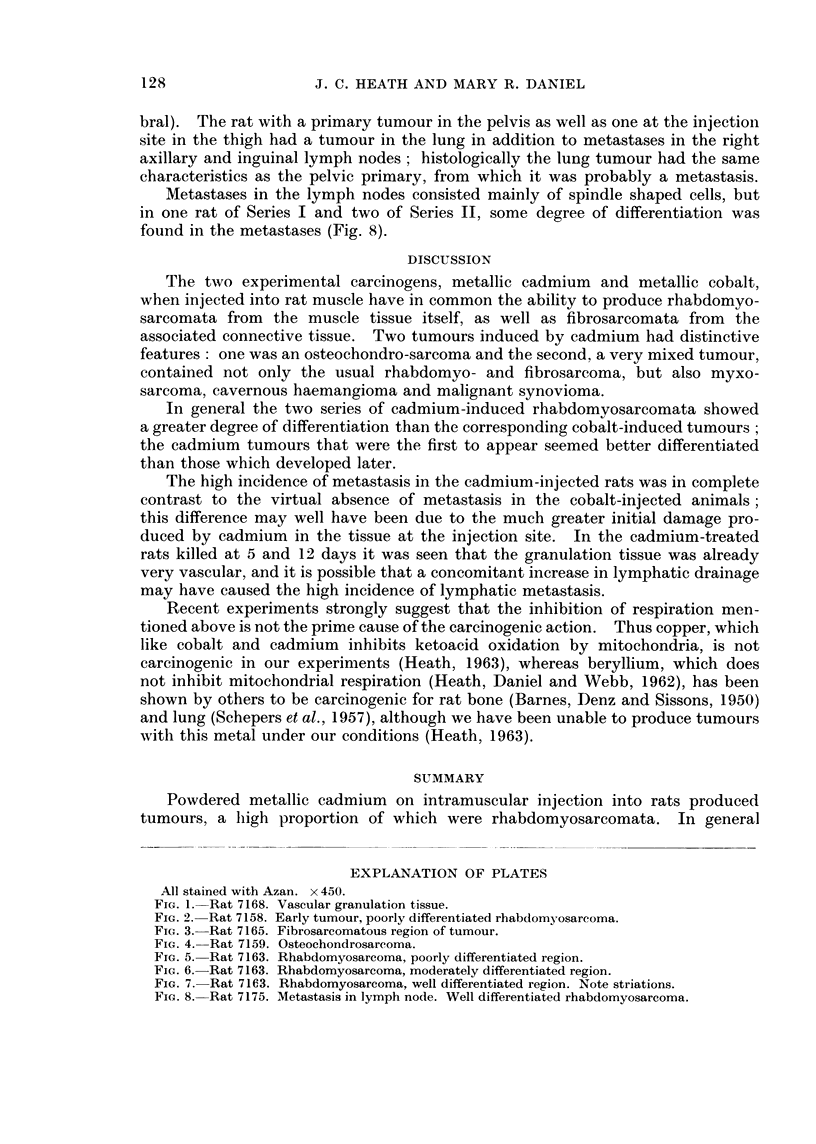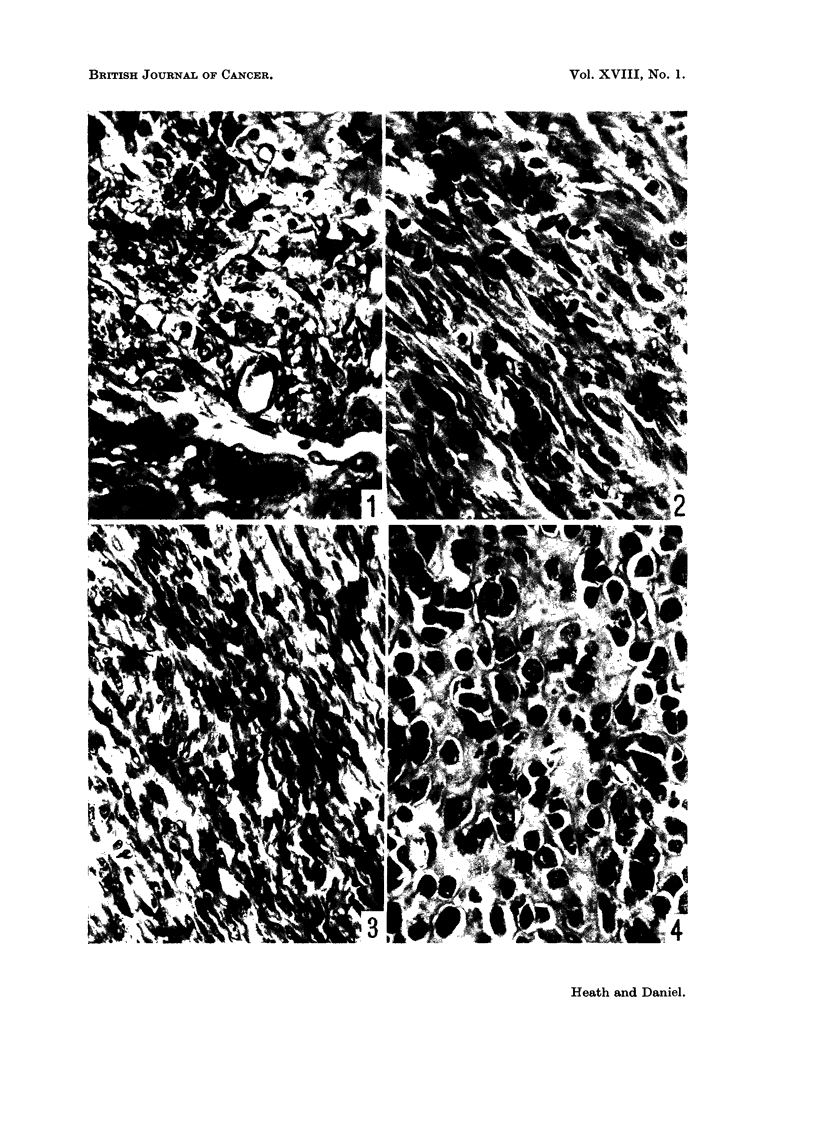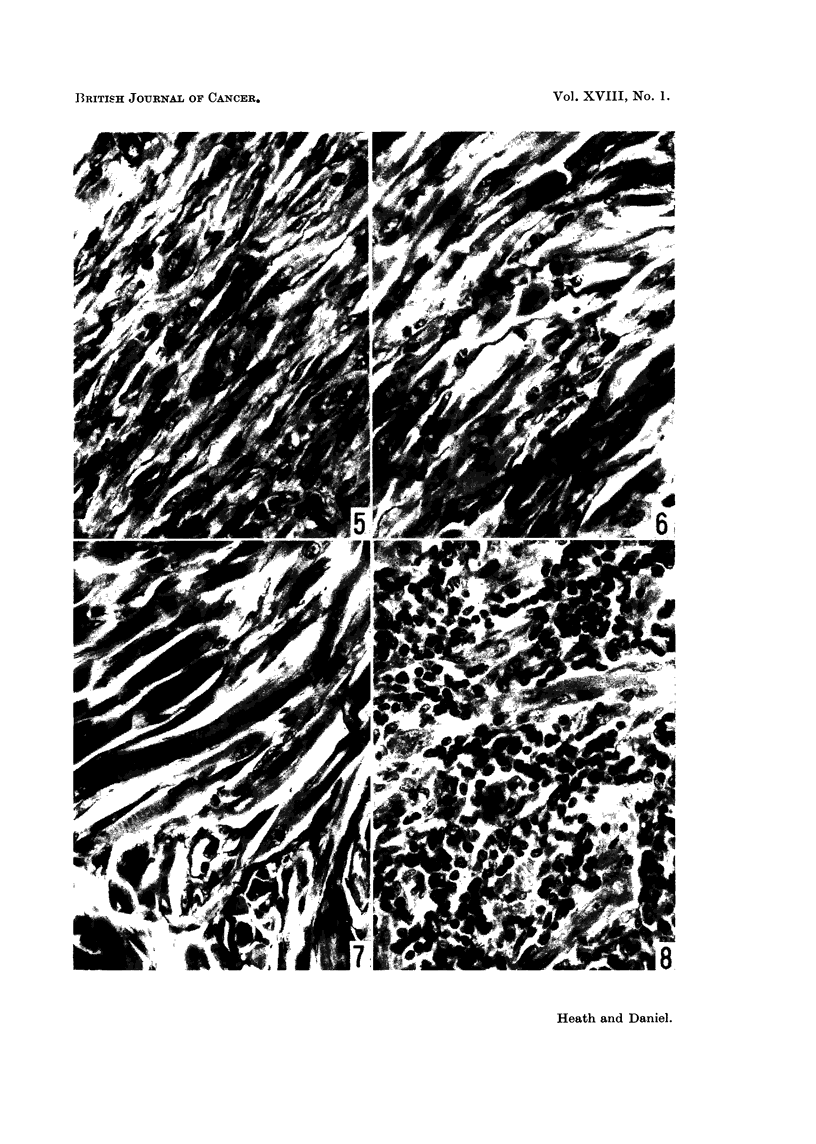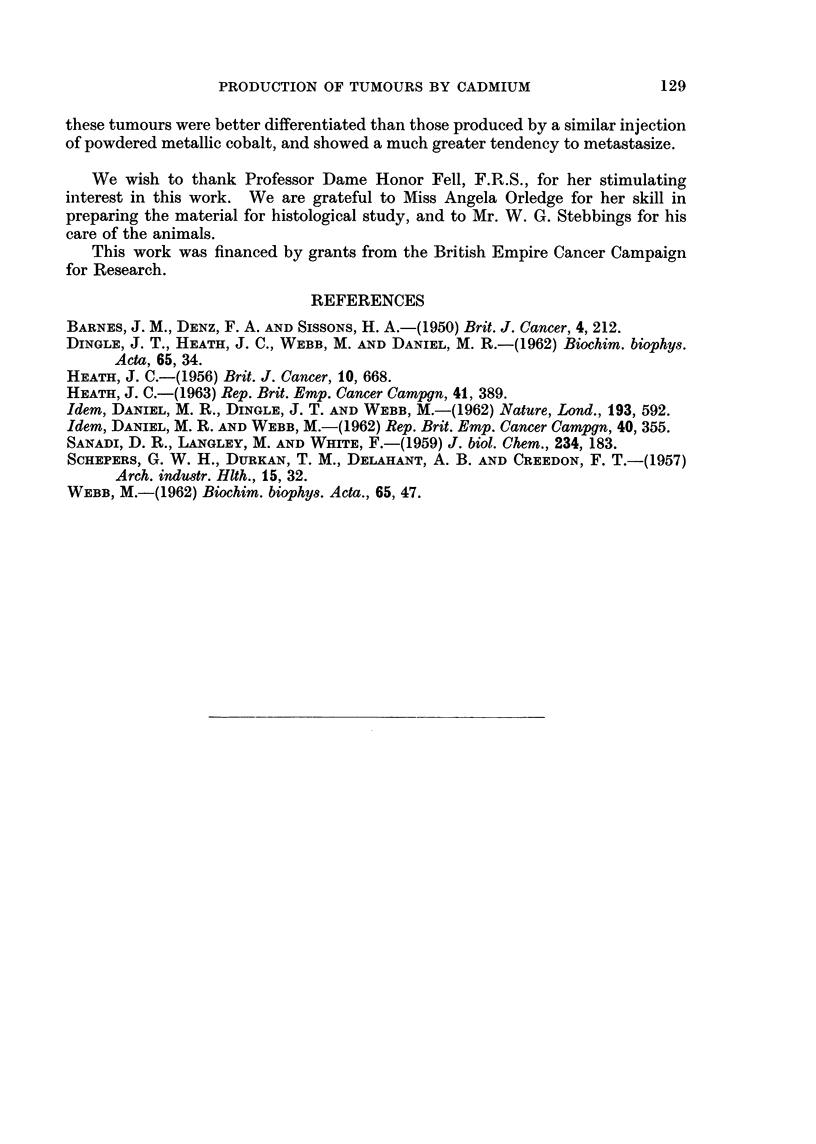# The Production of Malignant Tumours by Cadmium in the Rat

**DOI:** 10.1038/bjc.1964.13

**Published:** 1964-03

**Authors:** J. C. Heath, Mary R. Daniel

## Abstract

**Images:**


### THE PRODUCTION OF MALIGNANT TUMOURS BY CADMIUM IN THE RAT

J. C. HEATH AND MARY R. DANIEL

*From the Strangeways Research Laboratory, Cambridge*

Received for publication January 25, 1964

IN the search for the mechanism by which cobalt exerts its carcinogenic action, biochemical and tissue culture studies were made on this and other divalent metals (Cd, Ni, Cu, Hg, Zn, Be and Mn) to see (a) if any of the divalent metals which showed a similar metabolic action to that of cobalt was also carcinogenic (b) if the similar metabolic action of the carcinogenic metals was really related to the process of carcinogenesis.

The earlier work had shown that the respiration of mitochondria from rat liver and muscle was strongly inhibited by cobalt, the oxidations of pyruvate and $\alpha$-ketoglutarate being particularly susceptible. Sanadi, Langley and White (1959) had demonstrated an inhibition by cadmium of the enzymatic oxidation of $\alpha$-ketoglutarate ; as with cobalt (Dingle, Heath, Webb and Daniel, 1962) this effect appeared to be mediated through the formation of a complex with dihydro-lipoic acid (Webb, 1962). It seemed possible that the inhibition of respiration might be a mode of carcinogenesis. It was therefore logical to test cadmium for carcinogenicity by injection of the powdered metal into rat muscle as was done with cobalt (Heath, 1956). A high incidence of tumours at the injection site was obtained, and a preliminary report of the results has already appeared (Heath, Daniel, Dingle and Webb, 1962).

In the present paper these tumours are described in detail and compared with the cobalt-induced tumours previously investigated.

### MATERIALS AND METHODS

Two series of rats were injected on the same day ; in each series ten females of the hooded strain, age 2–3 months, were used. In Series I, 0·014 g. and in Series II 0·028 g. of cadmium metal powder (Hopkin and Williams, Ltd.) was shaken into suspension with 0·4 ml. of fowl serum and injected into the right thigh muscle of each animal from the medial aspect, approximately parallel with the femur and directed towards the hip. On microscopic examination the particles of metal were found to be of most varied shape including small and large spheres and ellipsoids, pyramids, rods and completely irregular forms. Dimensions ranged from 1·7 $\mu$ diameter (for spheres) to 85 $\mu \times 50 \mu$ for ellipsoids and rods, and 220 $\mu \times 50 \mu \times 50 \mu$ for the other shapes. Most of the particles were single.

### RESULTS

These are summarised in Tables I and II.

In every animal of both series injection of the cadmium was followed by an immediate severe local reaction, and after 3 days the injected thigh muscles were hard, swollen and tender. Histological examination of two animals of Series

II that were killed at 5 and 12 days respectively (Rats 7173 and 7168) showed that there was much necrosis with some attempt at repair ; the granulation tissue was very cellular and vascular (Fig. 1). This immediate severe reaction was in complete contrast to the early response to cobalt, which causes no clinical evidence of extensive damage, although microscopic examination reveals some necrosis. In the remaining cadmium-injected animals the reaction gradually subsided leaving the thigh muscles with contractures and much wasting ; the tumours subsequently developed in the wasted region.

*Incidence*

Nine of the ten rats of Series I and six of the eight (two were killed early) of Series II developed malignant tumours. The first positive malignant change was observed in a rat (No. 7158) of Series I which was killed at 13 weeks (Fig.2). Thereafter animals with tumours were killed at periods of $20\frac{1}{2}$ weeks to 56 weeks after injection ; the major dimensions of the tumours ranged from $2 \times 1\frac{1}{2} \times 1\frac{1}{2}$ cm. to $4\frac{1}{2} \times 4\frac{1}{2} \times 4$ cm. The remaining three rats were killed at 84 weeks with no apparent tumours ; both macroscopically and microscopically these animals all showed varying degrees of muscle wasting, but no evidence of malignant change.

Six animals of Series I and two of Series II had metastases in varying sites which included inguinal, prevertebral and axillary lymph nodes and lung. In one rat of Series I (No. 7159) there was a second primary tumour in the pelvic cavity.

*Gross appearance of tumours*

All but two tumours were firm, but the degree of hardness varied from tumour to tumour and indeed within the substance of the same tumour ; one was exceptionally hard. Some tumours contained localised gritty regions ; five showed some necrosis and haemorrhage. In two rats the bones of the leg showed thickening, some roughening of the surface and slight erosion.

*Histological appearance of tumours*

*Primary tumours.*—All the tumours had regions of rhabdomyosarcoma (Tables I and II), but six out of nine of Series I and three out of six of Series II also showed some areas of fibrosarcoma (Fig. 3). The tumour in rat 7169 had as additional components malignant synovioma, haemangioma and myxosarcoma, and the second primary in rat 7159 was composed of osteochondrosarcoma (Fig. 4) and rhabdomyosarcoma.

A distinctive feature of some of the tumours arising at the injection site was a great vascularity similar to, but less extensive than the definite haemangioma of the rat 7169.

In the two rats where the tumour had eroded the bone there was no evidence of malignant change in the bone surrounding the eroded region.

The degree of differentiation in the rhabdomyosarcomata varied both from animal to animal, and within a single tumour (Fig. 5–7). Some were much better differentiated than those produced by cobalt (Heath, 1956).

*Metastases.*—Six rats of Series I and two of Series II had metastatic deposits. All of the metastases from the primary tumours at the injection site in the leg were to lymph nodes (left and right inguinal, left and right axillary and preverte-

TABLE I—*Series I.*—0·014g. Cd/Rat

| Rat No. | Time to post-mortem (weeks) | Size and consistency of tumour | Type of tumour | Degree of differentiation | Sites of metastases | Histological type of metastases | Comments |
|---|---|---|---|---|---|---|---|
| 7158 | 13 | — | Rhabdomyosarcoma | Good | None | — | Very early tumour including evidence of pre-malignant change |
| 7159 | 37 | 3 × 4 × 4 cm.<br>Firm to hard<br>Soft to firm | Rhabdomyosarcoma<br>Fibrosarcoma | Poor to moderate<br>Poor to good | R axillary, R inguinal } lymph nodes | Poorly differentiated rhabdomyosarcoma | Second primary in pelvis—osteochondrosarcoma with metastasis in lung |
| 7160 | 37 | 3½ × 3 × 4 cm.<br>Very soft to soft | Rhabdomyosarcoma | Poor | None | — | Haemorrhagic area in tumour |
| 7161 | 48 | 4 × 3 × 4 cm.<br>Fairly soft | Rhabdomyosarcoma<br>Fibrosarcoma | Very poor | None | — | Rudimentary muscle fibre formation |
| 7162 | 84 | None | — | — | — | — | — |
| 7163 | 20½ | 3 × 3 × 3 cm.<br>Very hard : some fluid present | Rhabdomyosarcoma<br>Fibrosarcoma | Moderate to very good<br>Fair to moderate | Prevertebral, R & L axillary, R & L inguinal } lymph nodes | Undifferentiated | One area of very good differentiation—almost rhabdomyoma |
| 7164 | 39 | 3 × 2 × 2½ cm.<br>Firm | Rhabdomyosarcoma<br>Fibrosarcoma | Poor<br>Moderate to good | Prevertebral, R & L inguinal } lymph nodes | Poorly differentiated rhabdomyosarcoma | Some parts of rhabdomyosarcoma very vascular |
| 7165 | 39 | 2½ × 2½ × 2½ cm.<br>Firm | Rhabdomyosarcoma<br>Fibrosarcoma | Poor<br>Good | Prevertebral, R & L axillary, R & L inguinal } lymph nodes | Poorly differentiated rhabdomyosarcoma | Lymph node invasion myoblastic |
| 7166 | 51 | 3 × 1½ × 2½ cm.<br>Soft ;<br>haemorrhagic | Rhabdomyosarcoma | Poor | Prevertebral, R & L axillary, R & L inguinal } lymph nodes<br>Lung | Moderately differentiated rhabdomyosarcoma | Lymph node invasion myoblastic |
| 7167 | 56 | 4 × 4 × 4 cm.<br>Soft, with some "gritty" areas | Rhabdomyosarcoma<br>Fibrosarcoma | Poor<br>Poor | Prevertebral lymph nodes | Poorly differentiated rhabdo- and fibrosarcoma | Erosion of femur |

TABLE II.—*Series II*—0·028g. Cd/Rat

| Rat No. | Time to post-mortem (weeks) | Size and consistency of tumour | Type of tumour | Degree of differentiation | Sites of metastases | Histological type of metastases | Comments |
|---|---|---|---|---|---|---|---|
| 7168 | 12 days | — | — | — | — | — | Necrotic muscle with very cellular and vascular granulation tissue with some degeneration |
| 7169 | 32 | $2 \times 2\frac{1}{2} \times 2$ cm. Firm but not hard | Rhabdomyosarcoma<br>Fibrosarcoma<br>Malignant synovioma<br>Cavernous haemangioma<br>Myxosarcoma | Poorly differentiated rhabdomyosarcoma<br>Well differentiated fibrosarcoma | Prevertebral lymph nodes | Differentiated rhabdomyosarcoma | A very mixed tumour |
| 7170 | 24 | $4 \times 2\frac{1}{2} \times 2$ cm. Firm and gritty | Rhabdomyosarcoma | Moderate to good | None | — | Cystic degeneration of part of tumour |
| 7171 | 46 | $3 \times 5 \times 3$ cm. Soft and gelatinous | Rhabdomyosarcoma<br>Fibrosarcoma | Poor to medium | None | — | Fibrosarcoma a very minor component |
| 7172 | 84 | None | — | — | — | — | Normal muscle with some scar tissue—no sign of proliferation |
| 7173 | 5 days | None | — | — | — | — | Necrotic muscle with very cellular and vascular granulation tissue |
| 7174 | 23 | $4\frac{1}{2} \times 4\frac{1}{2} \times 4$ cm. Medium firm | Rhabdomyosarcoma | Very good | None | — | Solid tumour measured $2\frac{1}{2} \times 2\frac{1}{2} \times 2\frac{1}{2}$ cm. Remainder fluid (cystic degeneration) |
| 7175 | 29 | $3 \times 3 \times 2\frac{1}{2}$ cm. Firm, gristly | Rhabdomyosarcoma | Poor to moderate | R & L axillary, R & L inguinal, Prevertebral } lymph nodes | Very well differentiated rhabdomyosarcoma | Some regions of alveolar rhabdomyosarcoma. Some necrosis of bone |
| 7176 | 32 | $2 \times 1\frac{1}{2} \times 1\frac{1}{2}$ cm. Fairly soft to firm | Rhabdomyosarcoma<br>Fibrosarcoma | Both poorly differentiated | None | — | Tumour fairly vascular |
| 7177 | 84 | None | — | — | — | — | Atrophied. Much scar tissue. No proliferation |

bral). The rat with a primary tumour in the pelvis as well as one at the injection site in the thigh had a tumour in the lung in addition to metastases in the right axillary and inguinal lymph nodes ; histologically the lung tumour had the same characteristics as the pelvic primary, from which it was probably a metastasis.

Metastases in the lymph nodes consisted mainly of spindle shaped cells, but in one rat of Series I and two of Series II, some degree of differentiation was found in the metastases (Fig. 8).

### DISCUSSION

The two experimental carcinogens, metallic cadmium and metallic cobalt, when injected into rat muscle have in common the ability to produce rhabdomyosarcomata from the muscle tissue itself, as well as fibrosarcomata from the associated connective tissue. Two tumours induced by cadmium had distinctive features : one was an osteochondro-sarcoma and the second, a very mixed tumour, contained not only the usual rhabdomyo- and fibrosarcoma, but also myxosarcoma, cavernous haemangioma and malignant synovioma.

In general the two series of cadmium-induced rhabdomyosarcomata showed a greater degree of differentiation than the corresponding cobalt-induced tumours ; the cadmium tumours that were the first to appear seemed better differentiated than those which developed later.

The high incidence of metastasis in the cadmium-injected rats was in complete contrast to the virtual absence of metastasis in the cobalt-injected animals ; this difference may well have been due to the much greater initial damage produced by cadmium in the tissue at the injection site. In the cadmium-treated rats killed at 5 and 12 days it was seen that the granulation tissue was already very vascular, and it is possible that a concomitant increase in lymphatic drainage may have caused the high incidence of lymphatic metastasis.

Recent experiments strongly suggest that the inhibition of respiration mentioned above is not the prime cause of the carcinogenic action. Thus copper, which like cobalt and cadmium inhibits ketoacid oxidation by mitochondria, is not carcinogenic in our experiments (Heath, 1963), whereas beryllium, which does not inhibit mitochondrial respiration (Heath, Daniel and Webb, 1962), has been shown by others to be carcinogenic for rat bone (Barnes, Denz and Sissons, 1950) and lung (Schepers *et al.*, 1957), although we have been unable to produce tumours with this metal under our conditions (Heath, 1963).

### SUMMARY

Powdered metallic cadmium on intramuscular injection into rats produced tumours, a high proportion of which were rhabdomyosarcomata. In general

---

### EXPLANATION OF PLATES

All stained with Azan. ×450.

FIG. 1.—Rat 7168. Vascular granulation tissue.
FIG. 2.—Rat 7158. Early tumour, poorly differentiated rhabdomyosarcoma.
FIG. 3.—Rat 7165. Fibrosarcomatous region of tumour.
FIG. 4.—Rat 7159. Osteochondrosarcoma.
FIG. 5.—Rat 7163. Rhabdomyosarcoma, poorly differentiated region.
FIG. 6.—Rat 7163. Rhabdomyosarcoma, moderately differentiated region.
FIG. 7.—Rat 7163. Rhabdomyosarcoma, well differentiated region. Note striations.
FIG. 8.—Rat 7175. Metastasis in lymph node. Well differentiated rhabdomyosarcoma.

Heath and Daniel.

Heath and Daniel.

these tumours were better differentiated than those produced by a similar injection of powdered metallic cobalt, and showed a much greater tendency to metastasize.

We wish to thank Professor Dame Honor Fell, F.R.S., for her stimulating interest in this work. We are grateful to Miss Angela Orledge for her skill in preparing the material for histological study, and to Mr. W. G. Stebbings for his care of the animals.

This work was financed by grants from the British Empire Cancer Campaign for Research.

### REFERENCES

BARNES, J. M., DENZ, F. A. AND SISSONS, H. A.—(1950) *Brit. J. Cancer*, **4**, 212.

DINGLE, J. T., HEATH, J. C., WEBB, M. AND DANIEL, M. R.—(1962) *Biochim. biophys. Acta*, **65**, 34.

HEATH, J. C.—(1956) *Brit. J. Cancer*, **10**, 668.

HEATH, J. C.—(1963) *Rep. Brit. Emp. Cancer Campgn*, **41**, 389.

*Idem*, DANIEL, M. R., DINGLE, J. T. AND WEBB, M.—(1962) *Nature, Lond.*, **193**, 592.

*Idem*, DANIEL, M. R. AND WEBB, M.—(1962) *Rep. Brit. Emp. Cancer Campgn*, **40**, 355.

SANADI, D. R., LANGLEY, M. AND WHITE, F.—(1959) *J. biol. Chem.*, **234**, 183.

SCHEPERS, G. W. H., DURKAN, T. M., DELAHANT, A. B. AND CREEDON, F. T.—(1957) *Arch. industr. Hlth.*, **15**, 32.

WEBB, M.—(1962) *Biochim. biophys. Acta.*, **65**, 47.